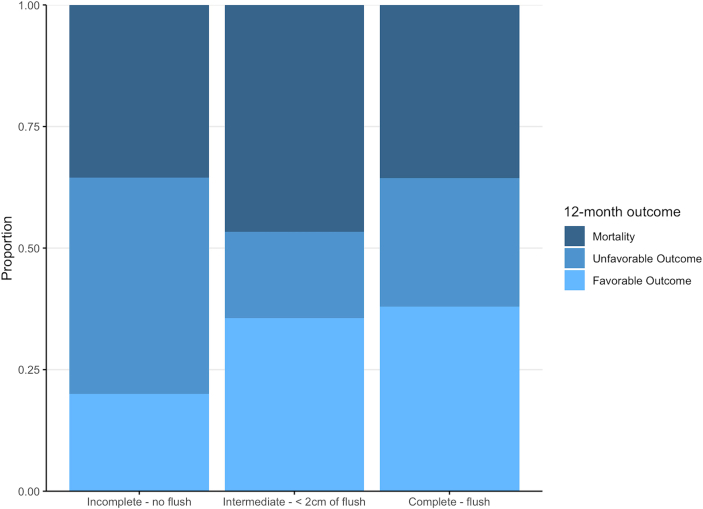# The effectiveness of decompressive craniectomy size in traumatic brain injury; an international, observational, comparative effectiveness study^☆^^☆☆^

**DOI:** 10.1016/j.bas.2026.106019

**Published:** 2026-04-03

**Authors:** Rick J.G. Vreeburg, Ranjit D. Singh, John K. Yue, Jeroen T.J.M. van Dijck, Hugo F. den Boogert, Jussi P. Posti, Wouter A. Moojen, Wilco C. Peul, Andrew I.R. Maas, Godard C.W. de Ruiter, Thomas A. van Essen, the CENTER-TBI participants and investigators, Cecilia Åkerlund, Krisztina Amrein, Nada Andelic, Lasse Andreassen, Audny Anke, Anna Antoni, Gérard Audibert, Philippe Azouvi, Maria Luisa Azzolini, Ronald Bartels, Pál Barzó, Romuald Beauvais, Ronny Beer, Bo-Michael Bellander, Antonio Belli, Habib Benali, Maurizio Berardino, Luigi Beretta, Morten Blaabjerg, Peter Bragge, Alexandra Brazinova, Vibeke Brinck, Joanne Brooker, Camilla Brorsson, Andras Buki, Monika Bullinger, Manuel Cabeleira, Alessio Caccioppola, Emiliana Calappi, Maria Rosa Calvi, Peter Cameron, Guillermo Carbayo Lozano, Marco Carbonara, Simona Cavallo, Giorgio Chevallard, Arturo Chieregato, Giuseppe Citerio, Hans Clusmann, Mark Coburn, Jonathan Coles, Jamie D. Cooper, Marta Correia, Amra Čović, Nicola Curry, Endre Czeiter, Marek Czosnyka, Claire Dahyot-Fizelier, Paul Dark, Helen Dawes, Véronique De Keyser, Vincent Degos, Francesco Della Corte, Hugo den Boogert, Bart Depreitere, Đula Đilvesi, Abhishek Dixit, Emma Donoghue, Jens Dreier, Guy-Loup Dulière, Ari Ercole, Patrick Esser, Erzsébet Ezer, Martin Fabricius, Valery L. Feigin, Kelly Foks, Shirin Frisvold, Alex Furmanov, Pablo Gagliardo, Damien Galanaud, Dashiell Gantner, Guoyi Gao, Pradeep George, Alexandre Ghuysen, Lelde Giga, Ben Glocker, Jagoš Golubovic, Pedro A. Gomez, Johannes Gratz, Benjamin Gravesteijn, Francesca Grossi, Russell L. Gruen, Deepak Gupta, Juanita A. Haagsma, Iain Haitsma, Raimund Helbok, Eirik Helseth, Lindsay Horton, Jilske Huijben, Peter J. Hutchinson, Bram Jacobs, Stefan Jankowski, Mike Jarrett, Ji-yao Jiang, Faye Johnson, Kelly Jones, Mladen Karan, Angelos G. Kolias, Erwin Kompanje, Daniel Kondziella, Evgenios Kornaropoulos, Lars-Owe Koskinen, Noémi Kovács, Ana Kowark, Alfonso Lagares, Linda Lanyon, Steven Laureys, Fiona Lecky, Didier Ledoux, Rolf Lefering, Valerie Legrand, Aurelie Lejeune, Leon Levi, Roger Lightfoot, Hester Lingsma, Andrew I.R. Maas, Ana M. Castaño-León, Marc Maegele, Marek Majdan, Alex Manara, Geoffrey Manley, Costanza Martino, Hugues Maréchal, Julia Mattern, Catherine McMahon, Béla Melegh, David Menon, Tomas Menovsky, Ana Mikolic, Benoit Misset, Visakh Muraleedharan, Lynnette Murray, Ancuta Negru, David Nelson, Virginia Newcombe, Daan Nieboer, József Nyirádi, Otesile Olubukola, Matej Oresic, Fabrizio Ortolano, Aarno Palotie, Paul M. Parizel, Jean-François Payen, Natascha Perera, Vincent Perlbarg, Paolo Persona, Wilco Peul, Anna Piippo-Karjalainen, Matti Pirinen, Dana Pisica, Horia Ples, Suzanne Polinder, Inigo Pomposo, Jussi P. Posti, Louis Puybasset, Andreea Radoi, Arminas Ragauskas, Rahul Raj, Malinka Rambadagalla, Isabel Retel Helmrich, Jonathan Rhodes, Sylvia Richardson, Sophie Richter, Samuli Ripatti, Saulius Rocka, Cecilie Roe, Olav Roise, Jonathan Rosand, Jeffrey V. Rosenfeld, Christina Rosenlund, Guy Rosenthal, Rolf Rossaint, Sandra Rossi, Daniel Rueckert, Martin Rusnák, Juan Sahuquillo, Oliver Sakowitz, Renan Sanchez-Porras, Janos Sandor, Nadine Schäfer, Silke Schmidt, Herbert Schoechl, Guus Schoonman, Rico Frederik Schou, Elisabeth Schwendenwein, Charlie Sewalt, Ranjit D. Singh, Toril Skandsen, Peter Smielewski, Abayomi Sorinola, Emmanuel Stamatakis, Simon Stanworth, Robert Stevens, William Stewart, Ewout W. Steyerberg, Nino Stocchetti, Nina Sundström, Riikka Takala, Viktória Tamás, Tomas Tamosuitis, Mark Steven Taylor, Aurore Thibaut, Braden Te Ao, Olli Tenovuo, Alice Theadom, Matt Thomas, Dick Tibboel, Marjolein Timmers, Christos Tolias, Tony Trapani, Cristina Maria Tudora, Andreas Unterberg, Peter Vajkoczy, Shirley Vallance, Egils Valeinis, Zoltán Vámos, Mathieu van der Jagt, Gregory Van der Steen, Joukje van der Naalt, Jeroen T.J.M. van Dijck, Inge A.M. van Erp, Thomas A. van Essen, Wim Van Hecke, Caroline van Heugten, Dominique Van Praag, Ernest van Veen, Thijs Van de Vyvere, Roel P.J. van Wijk, Alessia Vargiolu, Emmanuel Vega, Kimberley Velt, Jan Verheyden, Paul M. Vespa, Anne Vik, Rimantas Vilcinis, Victor Volovici, Nicole von Steinbüchel, Daphne Voormolen, Rick J.G. Vreeburg, Petar Vulekovic, Kevin K.W. Wang, Daniel Whitehouse, Eveline Wiegers, Guy Williams, Lindsay Wilson, Stefan Winzeck, Stefan Wolf, Zhihui Yang, Peter Ylén, Alexander Younsi, Frederick A. Zeiler, Veronika Zelinkova, Agate Ziverte, Tommaso Zoerle

**Affiliations:** f Department of Physiology and Pharmacology, Section of Perioperative Medicine and Intensive Care, Karolinska Institutet, Stockholm, Sweden; g János Szentágothai Research Centre, University of Pécs, Pécs, Hungary; h Division of Clinical Neuroscience, Department of Physical Medicine and Rehabilitation, Oslo University Hospital and University of Oslo, Oslo, Norway; i Department of Neurosurgery, University Hospital Northern Norway, Tromso, Norway; j Department of Physical Medicine and Rehabilitation, University Hospital Northern Norway, Tromso, Norway; k Trauma Surgery, Medical University Vienna, Vienna, Austria; l Department of Anesthesiology & Intensive Care, University Hospital Nancy, Nancy, France; m Raymond Poincare Hospital, Assistance Publique – Hopitaux de Paris, Paris, France; n Department of Anesthesiology & Intensive Care, S Raffaele University Hospital, Milan, Italy; o Department of Neurosurgery, Radboud University Medical Center, Nijmegen, the Netherlands; p Department of Neurosurgery, University of Szeged, Szeged, Hungary; q International Projects Management, ARTTIC, Munchen, Germany; r Department of Neurology, Neurological Intensive Care Unit, Medical University of Innsbruck, Innsbruck, Austria; s Department of Neurosurgery & Anesthesia & Intensive Care Medicine, Karolinska University Hospital, Stockholm, Sweden; t NIHR Surgical Reconstruction and Microbiology Research Centre, Birmingham, UK; u Anesthesie-Réanimation, Assistance Publique – Hopitaux de Paris, Paris, France; v Department of Anesthesia & ICU, AOU Città della Salute e della Scienza di Torino - Orthopedic and Trauma Center, Torino, Italy; w Department of Neurology, Odense University Hospital, Odense, Denmark; x BehaviourWorks Australia, Monash Sustainability Institute, Monash University, Victoria, Australia; y Department of Public Health, Faculty of Health Sciences and Social Work, Trnava University, Trnava, Slovakia; z Quesgen Systems Inc., Burlingame, CA, USA; aa Australian & New Zealand Intensive Care Research Centre, Department of Epidemiology and Preventive Medicine, School of Public Health and Preventive Medicine, Monash University, Melbourne, Australia; ab Department of Surgery and Perioperative Science, Umeå University, Umeå, Sweden; ac Department of Neurosurgery, Medical School, University of Pécs, Hungary and Neurotrauma Research Group, János Szentágothai Research Centre, University of Pécs, Hungary; ad Department of Medical Psychology, Universitätsklinikum Hamburg-Eppendorf, Hamburg, Germany; ae Brain Physics Lab, Division of Neurosurgery, Dept of Clinical Neurosciences, University of Cambridge, Addenbrooke's Hospital, Cambridge, UK; af Neuro ICU, Fondazione IRCCS Cà Granda Ospedale Maggiore Policlinico, Milan, Italy; ag ANZIC Research Centre, Monash University, Department of Epidemiology and Preventive Medicine, Melbourne, Victoria, Australia; ah Department of Neurosurgery, Hospital of Cruces, Bilbao, Spain; ai NeuroIntensive Care, Niguarda Hospital, Milan, Italy; aj School of Medicine and Surgery, Università Milano Bicocca, Milano, Italy; ak NeuroIntensive Care Unit, Department Neuroscience, IRCCS Fondazione San Gerardo dei Tintori, Monza, Italy; al Department of Neurosurgery, Medical Faculty RWTH Aachen University, Aachen, Germany; am Department of Anesthesiology and Intensive Care Medicine, University Hospital Bonn, Bonn, Germany; an Department of Anesthesia & Neurointensive Care, Cambridge University Hospital NHS Foundation Trust, Cambridge, UK; ao School of Public Health & PM, Monash University and the Alfred Hospital, Melbourne, Victoria, Australia; ap Radiology/MRI Department, MRC Cognition and Brain Sciences Unit, Cambridge, UK; aq Institute of Medical Psychology and Medical Sociology, Universitätsmedizin Göttingen, Göttingen, Germany; ar Oxford University Hospitals NHS Trust, Oxford, UK; as Intensive Care Unit, CHU Poitiers, Potiers, France; at University of Manchester NIHR Biomedical Research Centre, Critical Care Directorate, Salford Royal Hospital NHS Foundation Trust, Salford, UK; au Movement Science Group, Faculty of Health and Life Sciences, Oxford Brookes University, Oxford, UK; av Department of Neurosurgery, Antwerp University Hospital and University of Antwerp, Edegem, Belgium; aw Department of Anesthesia & Intensive Care, Maggiore Della Carità Hospital, Novara, Italy; ax Department of Neurosurgery, University Hospitals Leuven, Leuven, Belgium; ay Department of Neurosurgery, Clinical Centre of Vojvodina, Faculty of Medicine, University of Novi Sad, Novi Sad, Serbia; az Division of Anaesthesia, University of Cambridge, Addenbrooke's Hospital, Cambridge, UK; ba Center for Stroke Research Berlin, Charité – Universitätsmedizin Berlin, Corporate Member of Freie Universität Berlin, Humboldt-Universität zu Berlin, and Berlin Institute of Health, Berlin, Germany; bb Intensive Care Unit, CHR Citadelle, Liège, Belgium; bc Department of Anaesthesiology and Intensive Therapy, University of Pécs, Pécs, Hungary; bd Departments of Neurology, Clinical Neurophysiology and Neuroanesthesiology, Region Hovedstaden Rigshospitalet, Copenhagen, Denmark; be National Institute for Stroke and Applied Neurosciences, Faculty of Health and Environmental Studies, Auckland University of Technology, Auckland, New Zealand; bf Department of Neurology, Erasmus MC, Rotterdam, the Netherlands; bg Department of Anesthesiology and Intensive Care, University Hospital Northern Norway, Tromso, Norway; bh Department of Neurosurgery, Hadassah-hebrew University Medical Center, Jerusalem, Israel; bi Fundación Instituto Valenciano de Neurorrehabilitación (FIVAN), Valencia, Spain; bj Department of Neurosurgery, Shanghai Renji Hospital, Shanghai Jiaotong University/school of Medicine, Shanghai, China; bk Karolinska Institutet, INCF International Neuroinformatics Coordinating Facility, Stockholm, Sweden; bl Emergency Department, CHU, Liège, Belgium; bm Neurosurgery Clinic, Pauls Stradins Clinical University Hospital, Riga, Latvia; bn Department of Computing, Imperial College London, London, UK; bo Department of Neurosurgery, Hospital Universitario 12 de Octubre, Madrid, Spain; bp Department of Anesthesia, Critical Care and Pain Medicine, Medical University of Vienna, Austria; bq Department of Public Health, Erasmus Medical Center-University Medical Center, Rotterdam, the Netherlands; br College of Health and Medicine, Australian National University, Canberra, Australia; bs Department of Neurosurgery, Neurosciences Centre & JPN Apex Trauma Centre, All India Institute of Medical Sciences, New Delhi, 110029, India; bt Department of Neurosurgery, Erasmus MC, Rotterdam, the Netherlands; bu Department of Neurosurgery, Oslo University Hospital, Oslo, Norway; bv Division of Psychology, University of Stirling, Stirling, UK; bw Division of Neurosurgery, Department of Clinical Neurosciences, Addenbrooke's Hospital & University of Cambridge, Cambridge, UK; bx Department of Neurology, University of Groningen, University Medical Center Groningen, Groningen, the Netherlands; by Neurointensive Care, Sheffield Teaching Hospitals NHS Foundation Trust, Sheffield, UK; bz Salford Royal Hospital NHS Foundation Trust Acute Research Delivery Team, Salford, UK; ca Department of Intensive Care and Department of Ethics and Philosophy of Medicine, Erasmus Medical Center, Rotterdam, the Netherlands; cb Department of Clinical Neuroscience, Neurosurgery, Umeå University, Umeå, Sweden; cc Hungarian Brain Research Program - Grant No. KTIA_13_NAP-A-II/8, University of Pécs, Pécs, Hungary; cd Department of Anaesthesiology, University Hospital of Aachen, Aachen, Germany; ce Cyclotron Research Center, University of Liège, Liège, Belgium; cf Centre for Urgent and Emergency Care Research (CURE), Health Services Research Section, School of Health and Related Research (ScHARR), University of Sheffield, Sheffield, UK; cg Emergency Department, Salford Royal Hospital, Salford, UK; ch Institute of Research in Operative Medicine (IFOM), Witten/Herdecke University, Cologne, Germany; ci VP Global Project Management CNS, ICON, Paris, France; cj Department of Anesthesiology-Intensive Care, Lille University Hospital, Lille, France; ck Department of Neurosurgery, Rambam Medical Center, Haifa, Israel; cl Department of Anesthesiology & Intensive Care, University Hospitals Southhampton NHS Trust, Southhampton, UK; cm Cologne-Merheim Medical Center (CMMC), Department of Traumatology, Orthopedic Surgery and Sportmedicine, Witten/Herdecke University, Cologne, Germany; cn Intensive Care Unit, Southmead Hospital, Bristol, Bristol, UK; co Department of Neurological Surgery, University of California, San Francisco, CA, USA; cp Department of Anesthesia & Intensive Care, M. Bufalini Hospital, Cesena, Italy; cq Department of Neurosurgery, University Hospital Heidelberg, Heidelberg, Germany; cr Department of Neurosurgery, The Walton Centre NHS Foundation Trust, Liverpool, UK; cs Department of Medical Genetics, University of Pécs, Pécs, Hungary; ct Department of Neurosurgery, Emergency County Hospital Timisoara, Timisoara, Romania; cu School of Medical Sciences, Örebro University, Örebro, Sweden; cv Institute for Molecular Medicine Finland, University of Helsinki, Helsinki, Finland; cw Analytic and Translational Genetics Unit, Department of Medicine, Psychiatric & Neurodevelopmental Genetics Unit, Department of Psychiatry, Department of Neurology, Massachusetts General Hospital, Boston, MA, USA; cx Program in Medical and Population Genetics, The Stanley Center for Psychiatric Research, The Broad Institute of MIT and Harvard, Cambridge, MA, USA; cy Department of Radiology, University of Antwerp, Edegem, Belgium; cz Department of Anesthesiology & Intensive Care, University Hospital of Grenoble, Grenoble, France; da Department of Anesthesia & Intensive Care, Azienda Ospedaliera Università di Padova, Padova, Italy; db Dept. of Neurosurgery, Leiden University Medical Center, Leiden, the Netherlands; dc Dept. of Neurosurgery, Medical Center Haaglanden, The Hague, the Netherlands; dd Department of Neurosurgery, Helsinki University Central Hospital, the Netherlands; de Division of Clinical Neurosciences, Department of Neurosurgery and Turku Brain Injury Centre, Turku University Hospital and University of Turku, Turku, Finland; df Department of Anesthesiology and Critical Care, Pitié -Salpêtrière Teaching Hospital, Assistance Publique, Hôpitaux de Paris and University Pierre et Marie Curie, Paris, France; dg Neurotraumatology and Neurosurgery Research Unit (UNINN), Vall d'Hebron Research Institute, Barcelona, Spain; dh Department of Neurosurgery, Kaunas University of Technology and Vilnius University, Vilnius, Lithuania; di Department of Neurosurgery, Rezekne Hospital, Latvia; dj Department of Anaesthesia, Critical Care & Pain Medicine NHS Lothian & University of Edinburg, Edinburgh, UK; dk MRC Biostatistics Unit, Cambridge Institute of Public Health, Cambridge, UK; dl Department of Physical Medicine and Rehabilitation, Oslo University Hospital/University of Oslo, Oslo, Norway; dm Division of Orthopedics, Oslo University Hospital, Oslo, Norway; dn Institue of Clinical Medicine, Faculty of Medicine, University of Oslo, Oslo, Norway; do Broad Institute, Cambridge, MA, USA; dp Harvard Medical School, Boston, MA, USA; dq Massachusetts General Hospital, Boston, MA, USA; dr National Trauma Research Institute, The Alfred Hospital, Monash University, Melbourne, Victoria, Australia; ds Department of Neurosurgery, Odense University Hospital, Odense, Denmark; dt International Neurotrauma Research Organisation, Vienna, Austria; du Klinik für Neurochirurgie, Klinikum Ludwigsburg, Ludwigsburg, Germany; dv Division of Biostatistics and Epidemiology, Department of Preventive Medicine, University of Debrecen, Debrecen, Hungary; dw Department Health and Prevention, University Greifswald, Greifswald, Germany; dx Department of Anaesthesiology and Intensive Care, AUVA Trauma Hospital, Salzburg, Austria; dy Department of Neurology, Elisabeth-TweeSteden Ziekenhuis, Tilburg, the Netherlands; dz Department of Neuroanesthesia and Neurointensive Care, Odense University Hospital, Odense, Denmark; ea Department of Neuromedicine and Movement Science, Norwegian University of Science and Technology, NTNU, Trondheim, Norway; eb Department of Physical Medicine and Rehabilitation, St.Olavs Hospital, Trondheim University Hospital, Trondheim, Norway; ec Department of Neurosurgery, University of Pécs, Pécs, Hungary; ed Division of Neuroscience Critical Care, John Hopkins University School of Medicine, Baltimore, USA; ee Department of Neuropathology, Queen Elizabeth University Hospital and University of Glasgow, Glasgow, UK; ef Dept. of Department of Biomedical Data Sciences, Leiden University Medical Center, Leiden, the Netherlands; eg Department of Pathophysiology and Transplantation, Milan University, and Neuroscience ICU, Fondazione IRCCS Cà Granda Ospedale Maggiore Policlinico, Milano, Italy; eh Department of Radiation Sciences, Biomedical Engineering, Umeå University, Umeå, Sweden; ei Perioperative Services, Intensive Care Medicine and Pain Management, Turku University Hospital and University of Turku, Turku, Finland; ej Department of Neurosurgery, Kaunas University of Health Sciences, Kaunas, Lithuania; ek Intensive Care and Department of Pediatric Surgery, Erasmus Medical Center, Sophia Children's Hospital, Rotterdam, the Netherlands; el Department of Neurosurgery, Kings College London, London, UK; em Neurologie, Neurochirurgie und Psychiatrie, Charité – Universitätsmedizin Berlin, Berlin, Germany; en Department of Intensive Care Adults, Erasmus MC– University Medical Center Rotterdam, Rotterdam, the Netherlands; eo IcoMetrix NV, Leuven, Belgium; ep Psychology Department, Antwerp University Hospital, Edegem, Belgium; eq Director of Neurocritical Care, University of California, Los Angeles, USA; er Department of Neurosurgery, St.Olavs Hospital, Trondheim University Hospital, Trondheim, Norway; es Department of Emergency Medicine, University of Florida, Gainesville, FL, USA; et Department of Neurosurgery, Charité – Universitätsmedizin Berlin, Corporate Member of Freie Universität Berlin, Humboldt-Universität zu Berlin, and Berlin Institute of Health, Berlin, Germany; eu VTT Technical Research Centre, Tampere, Finland; ev Section of Neurosurgery, Department of Surgery, Rady Faculty of Health Sciences, University of Manitoba, Winnipeg, MB, Canada; a University Neurosurgical Center Holland, Leiden University Medical Center, Haaglanden Medical Center and Haga Teaching Hospital, Leiden and the Hague, the Netherlands; b Department of Neurosurgery, University of California, San Francisco, San Francisco, CA, USA; c Department of Neurosurgery and Turku Brain Injury Center, Turku University Hospital and University of Turku, Turku, Finland; d Department of Neurosurgery, Antwerp University Hospital, Edegem, Belgium; e Department of Translational Neuroscience, Faculty of Medicine and Health Science, University of Antwerp, Antwerp, Belgium

**Keywords:** Traumatic brain injury, Decompressive craniectomy, Size, CENTER-TBI, Large, Small

## Abstract

**Introduction:**

Guidelines recommend large decompressive craniectomies (DC) in traumatic brain injury (TBI), yet the optimal size remains debated. Real-world practice often differs from guideline recommendations and generalizability of prior evidence to a broader TBI population is uncertain.

**Research question:**

What is the comparative effectiveness of DC size on 12-month functional outcome in TBI?

**Material and methods:**

We selected participants enrolled in the CENTER-TBI diagnosed with TBI who received a hemicraniectomy. Effect of DC size on functional outcome was evaluated with random-effects logistic regression, associating center case-mix adjusted DC sizes to GOSE. Center preference was quantified with the median odds ratio (MOR).

**Results:**

Among 4509 patients enrolled in CENTER-TBI, 295 underwent a hemicraniectomy. DC size varied from 37 cm^2^ to 165cm^2^ (IQR 96 cm^2^-123cm^2^, ellipsoid calculation), with a two-times higher probability of receiving a 27 cm^2^ larger (IQR increase) DC for a similar patient in one center versus another random center (adjusted MOR for DC size 1·7). Only 4 patients received a DC ≥ 12 × 15 cm (cm) or 15 cm in diameter, while 0 patients received a DC ≤ 6 × 8 cm. Larger DC size was not associated with more favorable 12-month GOSE scores (aOR 0.73 for 27 cm2 increase in DC size, 95%CI 0.47-1.1).

**Discussion and conclusion:**

DC size varied widely across European centers. Recommended DC sizes were rarely reached, as were very small DC sizes. Larger versus smaller DC was associated with similar outcomes, however heterogeneity in DC indication may have attenuated observable treatment effects. Neurosurgeons may continue to prefer larger over smaller decompressions.

## Highlights

• Decompressive craniectomy (DC) size varied widely across European trauma centers.
• Guideline-recommended DC sizes were rarely reached in TBI patients.
• Larger DC size was not associated with improved 12-month functional outcome.

## 1 Background

Severe traumatic brain injury (TBI) remains a pressing global health concern, being the leading cause of injury related deaths and accounting for an estimated 82.000 annual deaths in Europe (James et al., 2019; Badhiwala et al., 2019). An important complication of severe TBI is the pathological increase of intracranial pressure (ICP) due to a complex interplay of different sequelae that are caused or worsened by extracranial and intracranial injuries (Stocchetti and Maas, 2014). An elevated ICP aggravates secondary brain injury by causing a mechanical shift of brain structures (herniation) and by disrupting cerebral blood flow and inducing ischemia (Hutchinson et al., 2013). Decompressive craniectomy (DC) may mitigate the deleterious effects of increased ICP (Timofeev et al., 2008). The procedure involves partial removal of the cranium and opening of the dura mater to allow brain swelling beyond the tabula interna (Quinn et al., 2011; Timofeev et al., 2012). DC can be performed as a primary (leaving the bone flap out after evacuation of a traumatic lesion) or secondary procedure (for refractory increased ICP) (Al-Jishi et al., 2011). It is usually performed as hemicraniectomy, but bifrontal or bilateral surgical approaches are also possible. Secondary DC is an effective last resort surgical treatment in lowering ICP and mortality in severe TBI (Cooper et al., 2011; Hutchinson et al., 2016; Kolias et al., 2022).

The intervention creates more space to compensate for the swollen brain, because there are less bony boundaries. Logically, the size of the DC may be relevant in this regard (Schur et al., 2020; Koo et al., 2021; Tanrikulu et al., 2015). Guidelines recommend to perform a large frontotemporoparietal DC of at least 12 × 15 cm or 15-cm diameter over a smaller frontotemporoparietal DC of 6 × 8 cm (Hutchinson et al., 2019; Hawryluk et al., 2020). These recommendations are primarily based on one randomized clinical trial (RCT) from 2005, but key limitations in this study raise concerns regarding the generalizability of its findings. In this RCT, the authors categorized DC sizes, which neglects more intermediate sizes and omits considerations of anatomical variations in skull sizes between TBI patients, the extent of temporal pole decompression, and the medial margin-to-midline distance, which could have contributed to patient outcome (Jiang et al., 2005). Furthermore, inclusion criteria were restricted to severe TBI patients with cerebral contusions or brain swelling, excluding patients over 70 years of age, patients with isolated acute subdural hematoma (ASDH) or epidural hematoma (EDH) and those initially presenting with mild or moderate TBI who later deteriorated.

These limitations and strict inclusion criteria highlight the need for a contemporary comparison of DC sizes to investigate if there are grounds for support of the recommended large 12 cm × 15 cm approach and to investigate if the recommendation is broadly applicable to a total TBI population. We therefore aimed to determine the effectiveness of DC size on functional outcome in TBI using the large dataset from the Collaborative European NeuroTrauma Effectiveness Research in TBI (CENTER-TBI).

## 2 Methods

### 2.1 Study design and population

This study utilizes data from the prospective, multicenter, observational cohort CENTER-TBI which enrolled participants from 65 centers across Europe between 2014 and 2017. CENTER-TBI is registered with ClinicalTrials.gov (NCT02210221). This study was predefined in a protocol and follows the Strengthening the Reporting of Observational Studies in Epidemiology (STROBE) statement (CENTER-TBI, 2024; Vandenbroucke et al., 2014; von Elm et al., 2007).

Participants who underwent a unilateral DC for a TBI are included in this study. The exclusion criteria were: (1) missing GOSE scores at 12 months after initial injury and (2) bifrontal or bilateral DC.

### 2.2 Definition of interventions

DC was defined as a partial removal of the cranium and opening of the dura mater to allow potential brain swelling beyond the tabula interna. The indication distinguishes between either a primary or secondary procedure. Primary DC was defined as the initial surgical evacuation of a traumatic mass lesion, followed by a per-operative decision not to reconstruct the cranium due to persistent intra-operative brain swelling or the suspicion that the initial TBI would secondarily lead to increasing brain swelling. Secondary DC was typically performed as a pre-emptive approach or as a last resort treatment of refractory ICP (van Essen et al., 2019).

The surgical technique and location of DC followed the treating neurosurgeon's preference, inherent to the observational nature of the study. Full temporal pole decompression was achieved if the bone margin of the decompression was flush with the middle cranial fossa (de Andrade et al., 2020; Lu et al., 2022). An intermediate pole decompression category was added representing a decompression of less than 2 cm of the temporal base. Opened mastoid air cells were defined as a DC, which extended into the mastoid air cells, in combination with hypopneumatisation. We defined medial margin-to-midline as the minimum distance between the medial margin of the DC and the midline of the brain (Beucler, 2023; Williams et al., 2021).

The maximum anterior-posterior (AP) DC length, the maximum rostral-caudal (RC) distance of the DC, the extent of temporal decompression and medial margin-to-midline distance was measured using the first post-operative CT scan. RC distances are perpendicular to the axial plane of the CT scan (Supplemental Fig. 1). The surface area of the DC (hence referred to as DC size) was estimated using the formula of an ellipse (*π∗a∗b*), where *a* equaled half the maximum AP DC length and *b* equaled half the DC width (Supplemental Fig. 1). The DC width was measured using Pythagoras’ theorem (*a*^*2*^ *+ b*^*2*^ *= c*^*2*^) where *a* equaled the distance between the lateral DC margin and the midline at the most caudal point of the DC and *b* equaled the RC distance of the DC (Supplemental Fig. 1). (Jägersberg et al., 2021)

The skull-adjusted bone flap measurements equaled the ratio of the flap circumference to the contralateral skull hemicircumference, calculated as (2π √(a^2^ + b^2^)/2)/2, where *a* was equal to ½ of the AP diameter and *b* was equal to ½ of the transverse diameter (Schur et al., 2020). The flap circumference was calculated using the elliptical arc equation √(Δx^2^ + Δy^2^) (Schur et al., 2020).

### 2.3 Outcomes

The primary outcome was the (derived) GOS-E collected at 12 months after the initial injury. The secondary outcomes are (1) GOS-E at 3 and 6 months after injury, (2) dichotomization of the GOS-E in favorable outcome (GOS-E 5-8, namely moderate to good recovery), (3) DC-related complications, such as transcalvarial brain herniation (‘mushroom’ phenomena), paradoxical herniation and post-traumatic hydrocephalus (PTH), (4) difference (Δ) in ICP between pre-operative and post-operative and (5) the descriptive comparison of skull-adjusted versus absolute classification of DC size. Transcalvarial brain herniation refers to the swelling of intracranial tissue beyond the tabula externa, visible on the first post-operative CT-scan (Riveros et al., 2019; Silva Neto and Valença, 2019). Sunken flap was diagnosed radiologically when there was a marked concavity of the overlying skin flap passing the tabula interna, e.g. paradoxical herniation (Santander et al., 2022; Vasung et al., 2016). PTH was diagnosed radiologically on the first post-operative CT-scan when ventriculomegaly was present, using pre-operative scans as comparison in cases of unclear distension of cerebral ventricles (Rekate, 2009).

### 2.4 Statistical analysis

In the main analysis, we used regression modelling using random-effects proportional odds ordinal regression. The independent variable was DC size (continuous) and the dependent variable was GOSE (ordinal). Age, baseline Glasgow Coma Score (GCS), pupil reactivity, if any major extracranial intervention was performed during ED admission and the presence of certain CT imaging variables, such as acute subdural hematoma (ASDH), epidural hematoma (EDH), intracerebral hemorrhage/contusions (ICH) and midline shift, were considered confounders and added as independent variables in the regression models (van Essen et al., 2020; Vreeburg et al., 2025a). The results of the random-effects regression yielded point-estimations for adjusted odds ratios (aOR) and corresponding 95% confidence intervals (CI). Adjusted odds ratios indicate the odds per 1-point incremental increase in GOS-E where larger DC sizes are compared to small ones.

Secondary analysis consisted of regression modelling using patient-level random-effects logistic regression and instrumental variable (IV) analysis for the extent of temporal decompression and medial margin-to-midline distance. The ΔICP between pre-operative and post-operative was analyzed using random-effects linear regression and IV analysis. Patient-level random-effect logistic regression (DC related complications) is employed for other secondary outcomes, adjusting for the same confounders from the primary analysis. Descriptive skull-adjusted versus absolute DC size comparisons are made based on the relative classification of small, intermediate and large using the IQR. Certain patients received a small absolute decompression which could be considered large relative to the skull and vice versa.

Furthermore, sensitivity analyses are performed using propensity score matching (PSM) with balanced parallel groups (1:1) using a nearest-neighbor approach with a caliper of 0.16. The PSM model contained the same independent variables as the primary random-effects logistic regression model. Besides patient-level analysis, outcomes were also evaluated with respect to center treatment strategy (and not actual treatment) using IV analyses (van Essen et al., 2020; Cnossen et al., 2018). This approach accounts for unmeasured confounding by using center-level variation in DC size preference as a surrogate for treatment assignment in the absence of randomization. Specifically, these analyses are a comparison of centers with different practices in DC size, quantified by the case-mix-adjusted probability of undergoing a larger DC at each participating center. Thus, centers are ranked based on their preferences for performing a larger DC, adjusted for patient-level characteristics, imaging findings and injury severity. The case-mix-adjusted probability of undergoing a larger DC at each participating center was calculated using linear regression with the same independent variables as the primary random-effects logistic regression. The association of the instrument (participating centers) with the treatment (DC size) is quantified using the median odds ratio (MOR). The MOR gauges treatment variation between centers that is not attributable to chance, nor explained by other variables, such as case mix. (Austin and Merlo, 2017; Merlo et al., 2006). We excluded centers with less than 8 patients to accurately assess a hospital's tendency to prefer larger or smaller DC size. To compare baseline characteristics and DC descriptives across DC sizes, patients are stratified according to the IQR for DC size.

Demographic data, injury characteristics and severity, CT imaging variables and DC descriptives are displayed using percentages, medians with interquartile ranges (IQR), standardized mean differences (SMD) and p-values. The statistical tests are based on the type and normality of the data.

Statistical analysis was performed using R version 4.4.0. Missing data were multiply imputed (n = 10) with a nearest neighbor approach using the ‘mice’ package (Austin et al., 2021). Data were accessed with a bespoke data management tool, Neurobot (research resource identifier: SCR_01700).

### 2.5 Ethics and approval

The CENTER-TBI study (EC grant 602150) has been conducted in accordance with all relevant laws of the EU and all relevant laws of the country where the recruiting sites were located. Informed Consent by the patients and/or legal representative was obtained according to local legislation. Ethical approval was obtained for each recruiting site. The list of site, ethical committees and approval numbers can be found on the CENTER-TBI website (CENTER-TBI, 2025).

## 3 Results

### 3.1 Baseline characteristics and DC descriptives

The CENTER-TBI core study included 4509 patients with TBI, of whom 326 underwent DC as a treatment for their TBI. Of those 326 DC patients, 27 patients received a bifrontal DC and 4 patients a bilateral DC. Accordingly, 295 patients were available for primary and secondary outcome analysis. DC size was illustratively stratified according to the IQR for DC size (Fig. 1, Fig. 2 & Supplemental Fig. 2). Patient baseline, injury and CT-imaging characteristics were generally similar across DC sizes, with only modest, statistically non-significant differences in age and pupillary reactivity (Table 1 & Supplemental Table 1). The DC location and type were similar between the size groups (Table 2). However, only a small proportion of DCs were performed as planned procedures for refractory ICP (19%), whereas the majority of DCs were either unplanned decisions due to intraoperative swelling or routinely performed during mass lesion evacuation (25% and 27%, respectively, Table 2). The skull-adjusted DC ratio in percentages was greater in the large DC group (68, IQR 64, 74] compared to the small (52, IQR 46-59) and intermediate DC sizes (60, IQR 55-65, Table 2). Medial margin-to-midline distance (centimeter [cm]) differed significantly between small, intermediate and large DC sizes (2.1 [IQR 1.4-2.8], 1.7 [IQR 1.2-2] and 1.1 [IQR 0.7-1.7], respectively). Full temporal pole decompression was more frequent in the large DC size group (n = 30/39, 77%) compared to the small (n = 12/38, 32%) and intermediate DC size groups (n = 37/81, 46%). The size of the DC varied from 37 cm^2^ to 165 cm^2^ (IQR 96 cm^2^-123cm^2^) between centers, with an almost two-times higher probability of receiving a 27 cm2 larger (IQR increase) for an identical patient in one center versus another center at random (adjusted MOR for acute surgery 1·7, Fig. 3). The MOR for intercountry random-effects variance was 1·7. Only 4 patients received a DC of at least 12 cm × 15 cm or 15 cm in diameter and 0 patients received a DC ≤ 6 cm × 8 cm.

**Fig. 1:**
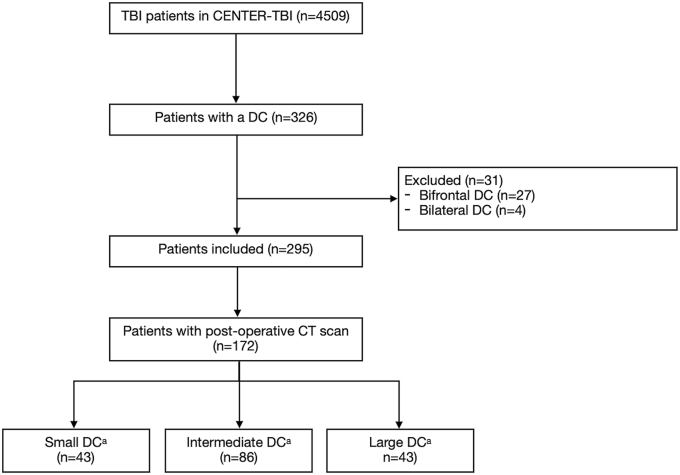
**Flow diagram of study population**. *Abbreviations:* CENTER-TBI, the Collaborative European NeuroTrauma Effectiveness Research in Traumatic Brain Injury; CT, computed tomography; DC, Decompressive craniectomy; n, number of patients. ^a^ DC size ≤ 95 cm^2^ (quantile 1) was labeled as small and a DC size ≥ 123 cm^2^ (quantile 3) was considered large. DC sizes between 95 cm^2^ and 123 cm^2^ were considered intermediate.

**Fig. 2:**
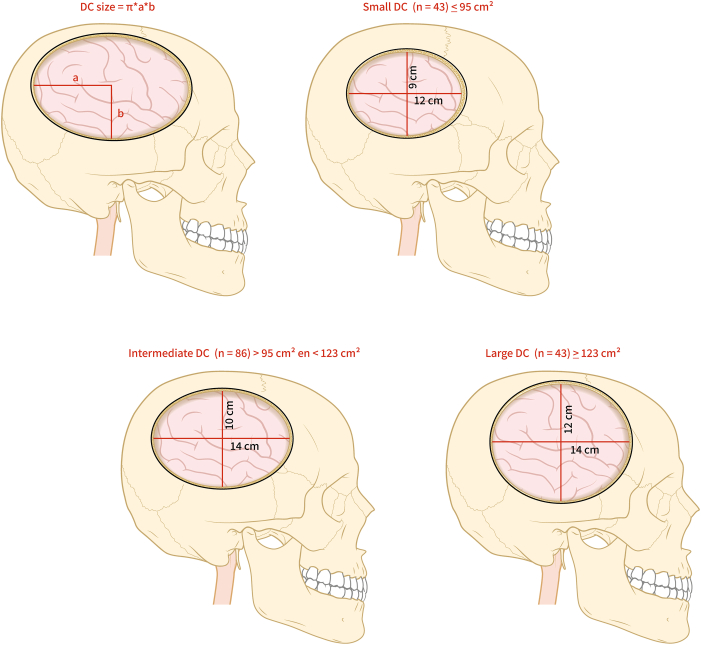
**Illustrative sagittal representation of DC sizes, anatomical positions and corresponding AP and RC distances**.^a^*Abbreviations:* AP, anteroposterior; cm, centimeter; cm^2^, cubic centimeter; DC, Decompressive craniectomy; n, number of patients; RC, rostrocaudal. ^a^ DC size ≤ 95 cm^2^ (quantile 1) was labeled as small and a DC size ≥ 123 cm^2^ (quantile 3) was considered large. DC sizes between 95 cm^2^ and 123 cm^2^ were considered intermediate. Please refer to the methods section for detailed calculation and rationale of DC sizes.

**Table 1:** Baseline characteristics of study population.

	Small	Intermediate	Large	p-value	SMD	Missing (%)
**No. of patients**	43	86	43			
**Age in years (median [IQR])**	56 [44, 63]	46 [30, 56]	36 [28, 59]	0.075	0.342	1.4
**Male sex (%)**	29 (67)	66 (78)	37 (86)	0.12	0.3	1.4
**GCS score (median [IQR])**	5 [3, 8]	5 [3, 9]	3 [3, 8]	0.37	0.16	7.5
**GCS motor score (median [IQR])**	2 [1, 5]	1 [1, 5]	1 [1, 4]	0.45	0.18	4.4
**Severity of TBI (%)**				0.77	0.21	7.5
Mild, GCS score 13-15	5 (12)	12 (15)	3 (8)			
Moderate, GCS score 9-12	3 (7)	9 (11)	5 (13)			
Severe, GCS score <9	33 (81)	60 (74)	30 (79)			
**Pupils (%)**				0.05	0.45	7.1
Both reacting	30 (73)	44 (54)	25 (64)			
One reacting	4 (10)	28 (35)	11 (28)			
Both unreactive	7 (17)	9 (11)	3 (8)			
**ASA status (%)**				0.81	0.16	9.2
I, A normal healthy patient	22 (52)	51 (63)	20 (56)			
II, A patient with mild systemic disease	14 (33)	21 (26)	12 (33)			
III, A patient with severe systemic disease	6 (14)	9 (11)	4 (11)			
**Anti-thrombotic usage (%)**				0.4	0.34	9.5
None	33 (81)	72 (88)	34 (94)			
Anticoagulants	3 (7)	2 (2)	0 (0)			
Platelet aggregation inhibitors	5 (12)	8 (9)	2 (6)			
**Cause of injury (%)**				0.69	0.34	12
Road traffic incident	14 (33)	30 (39)	17 (44)			
Incidental fall	22 (52)	39 (51)	16 (41)			
Other non-intentional injury	2 (5)	2 (3)	1 (3)			
Violence/assault	1 (2)	4 (5)	4 (10)			
Suicide attempt	3 (7)	2 (3)	1 (3)			
**Type of injury (%)**				0.3	0.4	4.7
Closed	41 (95)	79 (94)	34 (81)			
Blast	0 (0)	1 (1)	0 (0)			
Crush	0 (0)	1 (1)	1 (2)			
Penetrating-perforating	0 (0)	1 (1)	2 (5)			
Penetrating-tangential	0 (0)	0 (0)	1 (2)			
Closed with open depressed skull fracture	2 (5)	2 (2)	4 (10)			
**Epidural hematoma (%)**	6 (15)	11 (14)	8 (21)	0.6	0.13	8.5
**Acute subdural hematoma (%)**	18 (44)	42 (53)	21 (54)	0.58	0.13	7.9
**Cerebral contusions (%)**	21 (51)	37 (47)	24 (62)	0.35	0.19	8.5
**Skull fracture (%)**	8 (20)	16 (20)	6 (16)	0.84	0.08	8.5
**Midline shift (%)**^b^	32 (74)	63 (74)	33 (77)	0.95	0.04	0.6
**Diffuse axonal injury (%)**	3 (7)	9 (11)	7 (18)	0.31	0.23	8.5
**ED ventilation (%)**	22 (52)	36 (47)	20 (54)	0.76	0.09	10.2
**ED intubation (%)**	22 (52)	40 (52)	24 (63)	0.49	0.15	9.0
**Major ECI**^a^	13 (27)	24 (28)	12 (29)	0.986	0.09	0
**ICP monitoring during hospital admission (%)**	39 (91)	80 (94)	38 (88)	0.51	0.14	1.4
**IMPACT Unfavorable Outcome (median [IQR])**^c^	63 [47, 78]	67 [43, 78]	70 [52, 76]	0.94	0.06	32
**IMPACT Mortality (median [IQR])**^c^	45 [30, 63]	52 [28, 64]	50 [36, 64]	0.77	0.11	32
**Hospital length Of stay (median [IQR])**	31 [13, 58]	26 [9.8, 52]	35 [14, 73]	0.615	0.077	3.4
**ICU length of stay (median [IQR])**	15 [7.9, 23]	14 [8, 22]	16 [9, 31]	0.481	0.194	5.1

*Abbreviations*: ASA, American Society of Anesthesiologists; ECI, extracranial injury; ED, emergency department; GCS, Glasgow Coma Scale; ICP, intracranial pressure; ICU, intensive care unit; IMPACT, the International Mission for Prognosis and Analysis of Clinical Trials in Traumatic Brain Injury; IQR, interquartile range; SMD, standardized mean difference.

a An ECI requiring a hospital admission/intervention on its own, for example, external fixation of a limb, damage control thoracotomy, etc.

b Defined as midline shift more than 5 mm.

c Scores give the probability of an unfavorable outcome or death at 6 months postinjury. IMPACT probabilities only available in cases of GCS <13.

**Table 2:** Decompressive craniectomy descriptives.

	Small	Intermediate	Large	p	SMD	Missing (%)
**No. of patients**	43	86	43			
**DC AP length (median [IQR])**^a^	10 [9, 11]	12 [12, 13]	14 [13, 14]	<0.001	2.3	1.1
**DC rostral-caudal distance (median [IQR])**^a^	10 [9.7, 11]	12 [11, 12]	13 [12, 13]	<0.001	2.155	0.0
**DC minimal distance from midline (median [IQR])**^a^	2.1 [1.4, 2.8]	1.7 [1.2, 2]	1.1 [0.7, 1.7]	<0.001	0.771	1.7
**DC size (median [IQR])**^b^	83 [78, 90]	110 [104, 117]	136 [130, 142]	<0.001	3.597	0
**Proportion skull-adjusted DC in percentage (median [IQR])**	51 [46, 58]	60 [55, 65]	68 [64, 74]	<0.001	1.4	1.1
**Extent of temporal decompression**				<0.001	0.791	11
None (>2 cm of temporal skull base)	18 (47)	16 (20)	4 (10)			
Intermediate (<2 cm of temporal skull base)	8 (21)	28 (35)	5 (13)			
Full	12 (32)	37 (46)	30 (77)			
**Mastoid air cells opened**	3 (7)	5 (6)	7 (16)	0.121	0.225	0
**DC location (%)**				0.427	0.159	0
Right hemicraniectomy	26 (61)	54 (63)	22 (51)			
Left hemicraniectomy	17 (39)	32 (37)	21 (49)			
**DC type**				0.19	0.26	25
Primary	15 (58)	50 (76)	28 (76)			
Secondary	11 (42)	16 (24)	9 (24)			
**DC reason (%)**				0.311	0.541	7.9
Pre-emptive approach to treatment of (suspected) raised ICP (not last resort)	0 (0)	1 (1)	0 (0)			
Raised ICP, refractory to medical management (last resort)	8 (21)	14 (17)	9 (23)			
ICP not monitored, but CT evidence of raised ICP	14 (36)	15 (18)	9 (23)			
Not directly planned, but decided on because of intra-operative brain swelling	5 (13)	24 (29)	12 (30)			
Routinely performed with every ASDH or Contusion evacuation	9 (23)	25 (30)	10 (25)			
Development of cerebral infarction	3 (8)	5 (6)	0 (0)			
**DC procedure (%)**				0.744	0.254	8.5
Isolated procedure	8 (22)	12 (14)	6 (15)			
In association with ASDH removal	22 (58)	57 (67)	21 (54)			
In association with contusion/ICH removal	3 (7.9)	7 (8)	5 (13)			
In association with ASDH and contusion/ICH removal	5 (13)	9 (11)	7 (18)			

*Abbreviations:* AP, anterior-posterior; ASDH, acute subdural hematoma; cm, centimeter; CT, computed tomography; DC, decompressive craniectomy; ICH, intracerebral hemorrhage; ICP, intracranial pressure; IQR, interquartile range; No, number; SMD, standardized mean difference.

a In centimeters (cm).

b in square centimeters (cm^2^).

**Fig. 3:**
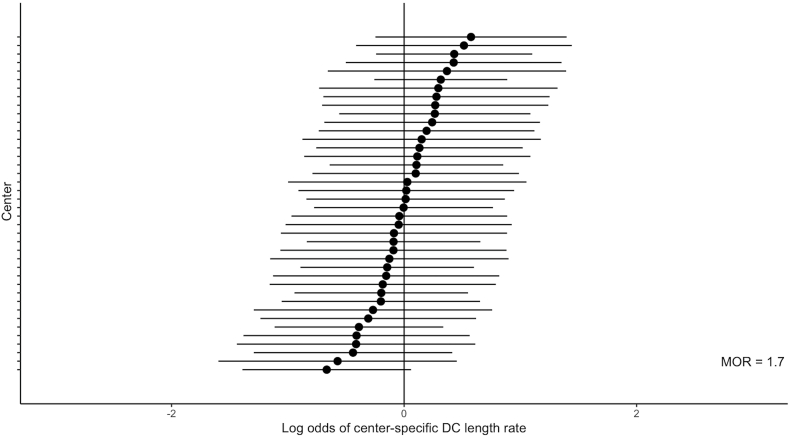
**Comparison of center-specific DC size rates**. The horizontal lines represent the log-odds of DC size rate of the individual participating centers (dot) and it’s corresponding 95% confidence interval (whiskers). *Abbreviations:* DC, decompressive craniectomy; MOR, median odds ratio.

Despite differences in baseline characteristics, the predicted outcomes of the IMPACT scores were similar across centers with varying surgical preferences for DC size (Supplemental Table 2), reflecting a balance in patient populations.

### 3.2 Outcomes

Larger DC size was not associated with an higher GOSE at 12 months (aOR 0.73 for 27 cm2 [IQR increase] larger DC, 95%CI 0.48-1.1, Table 3). Furthermore, extent of temporal decompression was also not associated with more favorable 12-month GOSE scores (aOR 1.2 for 27 cm2 larger DC, 95%CI 0.9-1.5). DC size was not correlated with higher proportions of favorable outcome (GOSE 5-8) when dichotomization the 12 month (aOR 0.79, 95%CI 0.49-1.4). Larger DCs were linked with lower 6-month GOSE scores (aOR 0.62 for 27 cm2 larger DC, 95%CI 0.38-0.89). Contrarily, larger distance between the medial margin of the DC and the midline was associated with higher 12-month GOSE (aOR 1.5 for 1.3 cm (IQR) increase in distance, 95% 1.1-2.1).

**Table 3:** | Associations of DC size with the primary outcome per IQR increase.

Outcome	Effect estimate	Adjusted (95% CI)
**12-month GOSE**		
Fixed-effects logistic regression model	OR	0.72 (0.48 – 1.1)
Random-effects logistic regression model	OR	0.73 (0.47 – 1.1)
Propensity score matching	OR	0.82 (0.48 – 1.4)
Instrumental variable	OR	0.75 (0.48 – 1.2)

*Abbreviations:* CI, Confidence Interval; DC, Decompressive craniectomy; GOSE, Glasgow Outcome Scale Extended; IQR, interquartile range; OR, odds ratio.

In random-effects linear regression, larger DC size was related to a greater decrease in post-operative ICP (β 6.3, 95%CI 3.6-8.9), but out of 125 patients for whom ICP measurements were recorded, only 35 received ICP monitoring before DC.

Transcalvarial brain herniation occurred in 39% (n = 17) of small, 55% (n = 49) of intermediate and 45% (n = 20) of large DCs, while sunken flap was present in 9% (n = 4/44) of small DC patients, 4% (n = 4/89) of intermediate and 4% (n = 2/44) of large DCs. The occurrence of PTH was higher in intermediate (n = 13, 15%) and large (n = 6, 14%) DC compared to small DC (n = 2, 4%). Using random-effects logistic regression, no associations were found regarding DC size and transcalvarial brain herniation (aOR 0.73, 95%CI 0.33-1.7), paradoxical herniation (aOR 1.1, 95%CI 0.72-1.7) and PTH (aOR 1.7, 95%CI 0.94-3, Table 4) diagnosed on the first available post-operative CT scan. Similarly, medial margin-to-midline distance was also not associated with the occurrence of PTH (aOR 0.75, 95%CI 0.36-1.6).

**Table 4:** | Associations of DC size (unless mentioned otherwise) with secondary outcomes per IQR increase including unadjusted odds ratios.

Outcome	Effect estimate	Unadjusted^a^ (95% CI)	Adjusted^b^ (95% CI)	IV-analysis (95% CI)
**6-month GOSE**	OR	0.72 (0.51 – 1)	0.62 (0.38 – 0.89)	0.8 (0.48 – 1.3)
**12-month GOSE**	OR	0.78 (0.53 – 1.2)	0.73 (0.48 – 1.1)	0.75 (0.48 – 1.2)
Extent of temporal decompression	OR	1.4 (1.1 – 1.7)	1.2 (0.9 – 1.5)	0.94 (0.53 – 1.7)
DC distance to midline (cm)	OR	1.7 (1.3 – 2.2)	1.5 (1.1 – 2.1)	1.2 (0.7 – 1.9)
Dichotomization – favorable outcome	OR	0.99 (0.61 – 1.6)	0.79 (0.49 – 1.4)	1.3 (0.89 – 1.9)
**ICP difference**^c^	β	5.9 (3.1 to 8.7)	6.3 (3.6 to 8.9)	−0.22 (−0.74 – 0.3)
**Complications**^d^				
Transcalvarial brain herniation	OR	1.1 (0.79 – 1.7)	0.73 (0.33 – 1.7)	e
Sunken flap/paradoxical herniation	OR	0.7 (0.32 – 1.5)	1.1 (0.72 – 1.7)	e
Post-traumatic hydrocephalus	OR	1.5 (0.94 – 2.3)	1.7 (0.94 – 3)	e

*Abbreviations:* β, Beta; CI, Confidence Interval; cm, centimeter; CT, computed tomography; DC, decompressive craniectomy; GCS, Glasgow Coma Scale, GOSE, Glasgow Outcome Scale Extended; ICP, intracranial pressure; IQR, interquartile range; IV, instrumental variable; OR, odds ratio.

a Unadjusted model consisted of a logistic ordinal regression model.

b Adjusted model consisted of a random-effect ordinal logistic regression. Age, pupillary reactivity, GCS at baseline, whether a major extracranial intervention was performed and the presence of subarachnoid hemorrhage, midline shift, acute subdural hematoma, epidural hematoma and cerebral contusions were considered confounders and added as independent predictors.

c ICP difference is calculated using the median ICP 24 h prior to DC till DC minus the median ICP from DC to 24 h after DC. A positive coefficient indicates a larger reduction in ICP as DC surface increases.

d Complications were diagnosed radiologically based on the first available post-operative CT.

e Unapplicable due to limited sample size, please refer to the limitations section of the discussion.

Disparities in classification between the absolute DC size and the skull-adjusted DC ratio were found (Supplemental Table 3). Most classification differences are between small absolute size to intermediate skull-adjusted ratio (n = 17), intermediate to small (n = 17), intermediate to large (n = 16) and large to intermediate (n = 15, Supplemental Table 3). Notable are the 4 DC's which were labeled as either small or large based on absolute DC size but large (n = 2) or small (n = 2), respectively, based on skull-adjusted DC ratio.

### 3.3 Sensitivity analysis

In a sensitivity analysis using PSM, no significant difference in 12-month GOSE scores was observed between DC sizes (aOR 0.82, 95%CI 0.48-1.4, Table 3). Moreover, center preference for a larger DC was not associated with better outcome according to GOSE at 12 months (aOR 0.75 for 27 cm2 [IQR increase] larger DC, 95%CI 0.48-1.2, Table 3). Similarly, in an IV analysis with dichotomization of the GOS-E, larger DC size was also not associated with higher odds of 12-month favorable outcome (aOR 1.3, 95%CI 0.89-1.9, Table 4). No association was found between DC size and GOS-E at 6 months after injury (Table 4). Furthermore, IV analysis demonstrated that larger medial margin-to-midline distance, greater extent of temporal decompression and skull-adjusted DC ratio were also not associated with 12-month GOSE (aOR 1.2, 95%CI 0.7-1.9 and aOR 0.94, 95%CI 0.53-1.7 and aOR 0.8, 95%CI 0.48-1.3, respectively, Table 4 & Supplemental Fig. 3).

In an IV analysis, larger DC size was not associated with a greater difference between pre- and post-operative ICP (β −0.22, 95%CI -0.74-0.3, Table 4).

## 4 Discussion

### 4.1 Summary of results

In this comparative effectiveness study, DC sizes varied substantially across centers. Larger DC size was not associated with more favorable 12-month GOSE scores, but the included DC represented a heterogenous group, including both planned decompressions for refractory ICP and cases where the bone flap was left out during mass lesion evacuation without explicit decompressive intent. Patients with TBI with similar characteristics were treated with different DC sizes because of surgical treatment preferences that varied across centers. Within this real-world practice context, patients treated in centers that used larger DCs did not have a better 12-month GOSE outcome than patients treated in centers that use smaller DCs. Also, greater extent of temporal decompression and greater medial margin-to-midline distance was not associated with higher GOSE scores.

### 4.2 Primary outcome

Larger DC size has been associated with a more favorable functional outcome after TBI when comparing large 15 cm × 12 cm to small 6 cm × 8 cm DC (Jiang et al., 2005). In our study, only four patients received a DC of 12 cm × 15 cm and zero patients a DC of 6 cm × 8 cm or smaller. This suggests that despite guideline recommendations based on good level evidence, the guideline adherence to create a sufficiently large DC in clinical practice is low. Nevertheless, the absence of patients receiving a DC smaller than 6 cm × 8 cm indicates adherence to the guideline. Notably, this contrasts with a 2005 report indicating that approximately up to one-third of DCs failed to achieve an adequate size (>30 cm^2^) (Compagnone et al., 2005). This may reflect a temporal trend toward larger decompressions and improved compliance with minimum size standards in contemporary practice. Nonetheless, these prior studies differed substantially in study design, inclusion criteria and underlying indications for DC, but do illustrate variability in DC sizing practices. Our study also mixed indications for DC and the observed variation in DC size must therefore be interpreted appropriately. In many cases, the intraoperative decision to leave the bone flap out may not have been driven by the goal of maximal decompression, but rather to avoid immediate replacement when uncertainty remained about possible postoperative swelling if the bone was replaced. This may have resulted in relatively small craniectomies that do not reflect intentional, guideline-directed DC and may have attenuated the apparent relationship between DC size and outcome.

We did find a correlation between DC size and 6-month GOSE but using IV analysis to adjust for potentially unmeasured confounding, our study did not confirm that larger DC size is associated with more favorable GOSE scores, even within the more commonly practiced DC sizes. Unlike conventional regression, IV analysis has the ability to address endogeneity by accounting for unmeasured confounding factors (Stel et al., 2012; Widding-Havneraas and Zachrisson, 2022). IV analysis uses an instrument, i.e. center treatment preference, that is correlated with DC size but not with outcome, to challenge unmeasured confounding. The observed differences in effect estimates between the adjusted random-effects logistic regression and IV analysis for certain secondary outcomes suggests that unmeasured confounding may have biased the conventional estimates. This highlights the importance of applying causal inference methods when residual confounding is a concern (Cnossen et al., 2018).

To further disentangle the effect of the DC size in a center from other between-center variations in care associated with outcome, the effect of the treatment strategy on outcome was modelled with adjustment for other between-center differences using a random effect for center (Cnossen et al., 2018). The absence of a clear treatment effect may reflect that the recommended DC sizes were rarely achieved in our study.

### 4.3 Secondary outcomes

Previous reports demonstrated that larger DC is associated with a greater difference between pre- and post-operative ICP (Kolias et al., 2022; Jiang et al., 2005). Larger cranial vaults immediately allow for more substantial outward expansion of intracranial tissue, especially in cases of generalized edema, thus lowering ICP more effectively compared to smaller DCs. We found similar results in the random-effects regression with greater decreases in post-operative ICP in larger decompressions, but we did not confirm these previous findings in a sensitivity analysis using IV. The lack of an association in the IV analysis may be attributed to the timing of ICP monitor placement. Most ICP monitors were placed concomitant with a DC and thus many pre-operative ICP measurements were missing, which may have influenced the IV analysis more than conventional random-effects regression (Boef et al., 2014).

Reports linking transcalvarial brain herniation to DC size are scarce. Our study found 86 cases of transcalvarial brain herniation, but its occurrence was not associated with DC size. The limited amount of transcalvarial brain herniation cases might be because a subset of DCs were not performed under conditions of malignant swelling, but often as routinely performed decompressions during mass lesion evacuation.

The risk of paradoxical herniation and PTH was similar across DC sizes. Paradoxical herniation, also known as sunken flap syndrome, has been previously associated with large DC sizes and a small medial margin-to-midline distance (<2 cm) (Di Rienzo et al., 2020). Our study did not confirm these findings, which may be due to inherent differences in radiological versus clinical diagnosing.

Greater skull-adjusted DC ratio (relative size) was also not associated with more favorable 12-month GOSE, in line with the results of the primary analysis on absolute sizes. Absolute and relative size classifications were largely concordant, with only 4 cases classified as small or large based on absolute size but reclassified as large or small, respectively, based on relative (skull-adjusted) DC size.

### 4.4 Targeted decompression

The effectiveness of a DC is not solely determined by DC size, but also by various other surgery-related factors. Limited medial margin-to-midline distance has been associated with an increased risk of subdural hygroma, although its relationship with post-traumatic hydrocephalus is controversial (Beucler, 2023; Williams et al., 2021; De Bonis et al., 2013). It is hypothesized that the PTH development after DC may be influenced by alterations in CSF dynamics, particularly during the venous reabsorption phase (Beucler, 2023; De Bonis et al., 2010, 2013). Smaller medial margin to the midline may further disrupts functional structure between bridging veins and venous sinuses of the dura mater, affecting the production and absorption of extracellular fluid dynamics (De Bonis et al., 2010; Anile et al., 2009; Nakagawa et al., 1974). However, we found no association of DC size or medial margin-to-midline distance with the risk of PTH. This might be due to differences in diagnostic criteria, with the current study relying on radiological assessment of PTH, in contrast to the combined clinical and radiological approach used in prior reports (Beucler, 2023; Williams et al., 2021; De Bonis et al., 2013; De Bonis et al., 2010; De Bonis and Anile, 2020).

### 4.5 Study strengths and limitations

Our study is the hitherto largest multicenter cohort study using prospective standardized data collection from over 65 centers across Europe, with predefined provider profiling.

Despite comparable baseline characteristics and prognosis, confounding by indication may persist. Nonetheless, our findings were robust since similar associations were found between random-effects regression with PSM and the estimates from IV analysis, indicative of limited bias from unmeasured confounding in the primary outcome (endogeneity). Endogeneity occurs when unmeasured confounders influence both the DC size and outcome, thus potentially biasing effect estimates. IV analysis addresses this by using an instrument, in our case center treatment strategy, which is uncorrelated with the error term and can therefore isolate the variation in DC size that is exogenous (Stel et al., 2012; Widding-Havneraas and Zachrisson, 2022). Nevertheless, due to a limited sample size and thus relatively low occurrence of certain complications, we were unable to perform an IV analysis for these safety outcomes. Furthermore, heterogeneity in patient characteristics and interventions (combining primary and secondary DC) remains a limitation, and our ability to perform subgroup analyses, particularly using IV analysis, was constrained by the limited sample size. The variation in DC indication limits the ability to attribute outcomes solely to DC size. In many cases, the bone flap was left out opportunistically during mass lesion evacuation rather than intentionally to achieve maximal decompression. Therefore, the observed treatment effect of DC size in our study may have been attenuated.

Surgeon-specific identifiers were not collected within the data. Therefore, center-level treatment preference served as a surrogate instrument, reflecting aggregated surgeon behaviors and institutional protocols (Vreeburg et al., 2025b). This approach is consistent with prior multicenter causal inference studies in TBI (Vreeburg et al., 2024; van Essen et al., 2022; Van Essen et al., 2025).

Unfortunately, we could only collect paradoxical herniation/sunken flap, transcalvarial herniation and PTH using radiological signs, limiting generalizability. Sunken flap syndrome, transcalvarial herniation and PTH should be appreciated as a clinical condition also (Santander et al., 2022; Gurjar et al., 2019). Our findings may therefore not represent the actual occurrence of sunken flap syndrome, transcalvarial herniation and PTH adequately. Also, controversy exists regarding the classification of transcalvarial herniation as a complication or a desirable, physiological process after DC to decrease ICP by allowing expansion of intracranial tissue outwards (Silva Neto and Valença, 2019; Gurjar et al., 2019; Liao et al., 2015).

Additionally, due to limitations in the data, we were only able to diagnose PTH on the first available post-operative CT-scan. PTH may develop weeks or even months after TBI and is potentially also influenced by the subsequent cranioplasty (De Bonis and Anile, 2020; Vreeburg et al., 2024). Given this prolonged timeframe, our measurements might have been premature. Unfortunately, many 1-, 2- or 3-month follow-up scans were not part of the mandated follow-up protocol of CENTER-TBI and therefore missing or not performed, which limited diagnostic options.

## 5 Conclusions

DC size varied widely across European centers. Recommended DC sizes were rarely reached, as were very small DC sizes. Larger versus smaller DC was associated with similar outcomes, however heterogeneity in DC indication may have attenuated observable treatment effects. Neurosurgeons may continue to prefer larger over smaller decompressions.

## Authorship contribution statement

R.J.G. Vreeburg: writing – original draft (lead), formal analysis (lead), Methodology (supporting); Visualization (lead); writing – review and editing (equal); data curation (equal). Ranjit D. Singh: writing – review and editing (equal); Methodology (supporting). John K. Yue: writing – review and editing (equal). Jeroen T.J.M. van Dijck: writing – review and editing (equal); Methodology (supporting). Hugo F. den Boogert; writing – review and editing, Methodology (supporting). Jussi P. Posti; writing – review and editing, Methodology (supporting). Wouter A. Moojen: writing – review and editing (equal); Methodology (supporting); Supervision (equal). Wilco C. Peul: Conceptualization (equal); writing – review and editing (equal); Supervision (equal). Andrew I.R. Maas: writing – review and editing (equal); Supervision (equal). Godard C.W. de Ruiter; writing – review and editing (equal); Supervision (equal). Thomas a. van Essen; Conceptualization (equal), writing – review and editing (equal); Methodology (supporting); Writing – original draft (supporting); Supervision (equal).

## Author disclosure statements

The authors declare no competing interest.

## Declaration of generative AI and AI-assisted technologies in the writing process

During the preparation of this work the author used Grammarly and ChatGPT as a final check in order to potentially improve readability and language.

## Funding

CENTER-TBI was supported by the European Union 7th Framework Programme for Research (grant no. 602150; A.I.R.M.), 10.13039/501100007731Hannelore Kohl Stiftung (Germany), and OneMind (United States). Patient travel and stipend expenses were supported by 10.13039/100018727One Mind (Staglin Family and General Peter Chiarelli). Dr. Yue reported grants from the 10.13039/100005351Neurosurgery Research and Education Foundation and Bagan Family Foundation Research Fellowship (award no. A139203, to the University of California, San Francisco) outside the submitted work. Dr. Maas reported grants from the European Union 7th Framework Programme for Research during the conduct of the study. Dr. Peul reported grants from European Committee Grant CENTER-TBI and grants from the Netherlands 10.13039/501100000942Brain Foundation during the conduct of the study. Dr. van Essen reported grants from the European Union 7th Framework Programme for Research or CENTER-TBI and the Niels Stensen Fellowship during the conduct of the study. The funders of the study had no role in study design, data collection, data analysis, data interpretation, or writing of the report.

## Figures and Tables

**figs1:**
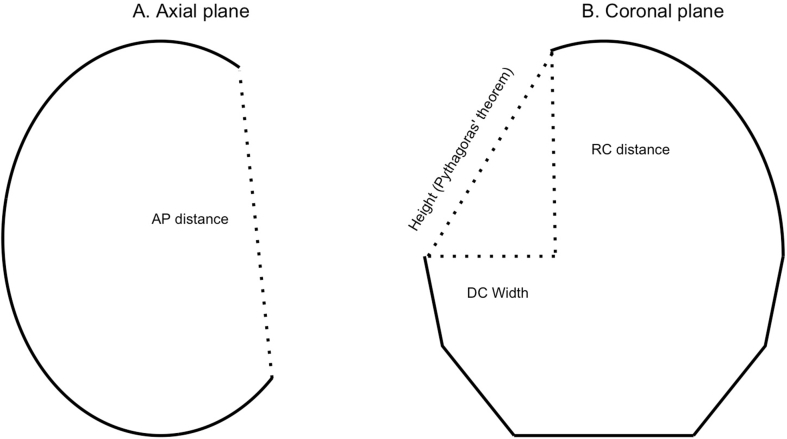


**figs2:**
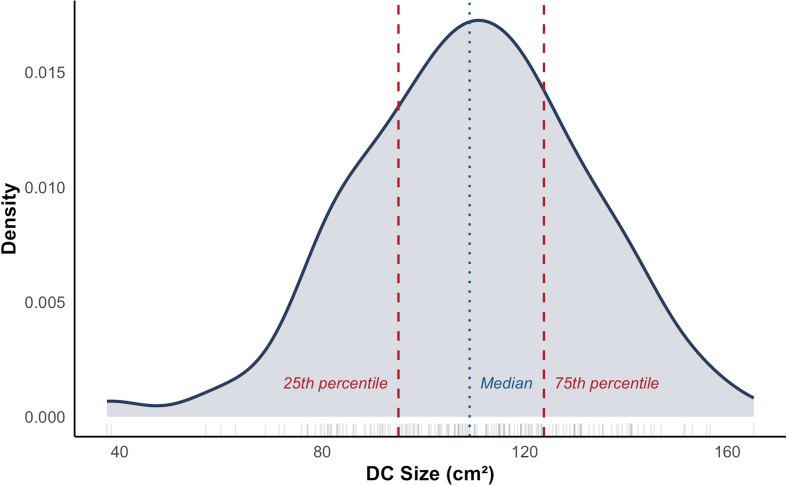


**figs3:**