# Supplementary material for: The effectiveness of decompressive craniectomy size in traumatic brain injury; an international, observational, comparative effectiveness study
Source: Brain Spine. 2026 Apr 3;6:106019. doi: 10.1016/j.bas.2026.106019 (PMC13090328; doi:10.1016/j.bas.2026.106019)
Supplement: Multimedia component 1 [file mmc1.docx]

**Supplemental Table 1 | Imaging lesion descriptives across the DC size groups in the core data**

|  | **Small** | **Intermediate** | **Large** | **p-value** | **SMD** |
| --- | --- | --- | --- | --- | --- |
| **No. of patients** | 43 | 86 | 43 |  |  |
| **Midline shift^a^ (%)** | 32 (75) | 63 (74) | 33 (77) | 0.95 | 0.04 |
| **Midline shift measure [median (IQR)]** | 9.5 (7 – 14) | 8 (7 – 13) | 7 (5 – 16) | 0.73 | 0.09 |
| **Acute subdural hematoma (%)** | 18 (44) | 42 (53) | 21 (54) | 0.58 | 0.13 |
| **Acute subdural hematoma maximum thickness in centimeters [median (IQR)]** | 0.86 (0.51 – 1.1) | 0.78 (0.5 – 1.1) | 0.84 (0.5 – 1.1) | 0.99 | 0.04 |
| **Acute subdural hematoma volume in milliliter^b^ [median (IQR)]** | 39 (21 – 70) | 43 (24 – 74) | 47 (17 – 72) | 0.9 | 0.1 |
| **Epidural hematoma (%)** | 6 (15) | 11 (14) | 8 (21) | 0.6 | 0.13 |
| **Epidural hematoma volume in milliliter^b^ [median (IQR)]** | 4.7 (1.8 – 16) | 16 (2.2 – 37) | 4.5 (1.3 – 8.7) | 0.2 | 0.6 |
| **Cerebral contusions (%)** | 21 (51) | 37 (47) | 24 (62) | 0.35 | 0.19 |
| **Cerebral contusions volume in milliliter^b^ [median (IQR)]** | 12 (5.4 – 25) | 11 (4.5 – 25) | 23 (11 – 38) | 0.15 | 0.26 |
| **Combined volume of intracranial lesions in milliliter^c^ [median (IQR)]** | 59 (41 – 87) | 57 (35 – 92) | 62 (28 – 106) | 0.94 | 0.06 |

*Abbreviations*: IQR, interquartile range; No, number; SMD, standardized mean difference

^a^Defined as midline shift more than 5 mm.

^b^Volume of the largest lesion

^c^Combined volume of ASDH, EDH and cerebral contusions/ICH
