# Supplementary material for: The effectiveness of decompressive craniectomy size in traumatic brain injury; an international, observational, comparative effectiveness study
Source: Brain Spine. 2026 Apr 3;6:106019. doi: 10.1016/j.bas.2026.106019 (PMC13090328; doi:10.1016/j.bas.2026.106019)
Supplement: Multimedia component 2 [file mmc2.docx]

**Supplemental Table 2 | Baseline characteristics of study population per center preference of DC size^a^**

|  | **Least likely** | **Less likely** | **More likely** | **Most Likely** | **p-value** |
| --- | --- | --- | --- | --- | --- |
| **No. of patients** | 57 | 48 | 47 | 41 |  |
| **Age in years (median [IQR])** | 51 [34, 60] | 45 [30, 56] | 39 [24, 55] | 42 [28, 58] | 0.29 |
| **GCS score (median [IQR])** | 8 [3, 11] | 6 [3, 8] | 7 [4, 13] | 6 [3, 14] | 0.42 |
| **GCS motor score (median [IQR])** | 4 [1, 6] | 2 [1, 5] | 4 [1, 6] | 3 [1, 6] | 0.44 |
| **Severity of TBI (%)** |  |  |  |  | 0.08 |
| Mild, GCS score 13-15 | 4 (12) | 3 (14) | 5 (19) | 8 (47) |  |
| Moderate, GCS score 9-12 | 13 (38) | 5 (23) | 8 (30) | 3 (18) |  |
| Severe, GCS score <9 | 17 (50) | 14 (64) | 14 (52) | 6 (35) |  |
| **Pupils (%)** |  |  |  |  | 0.02 |
| Both reacting | 32 (63) | 28 (67) | 24 (59) | 27 (79) |  |
| One reacting | 11 (22) | 2 (5) | 3 (7) | 1 (3) |  |
| Both unreactive | 8 (16) | 12 (29) | 14 (34) | 6 (18) |  |
| **ASA status (%)** |  |  |  |  | <0.001 |
| I, A normal healthy patient | 31 (57) | 17 (40) | 36 (88) | 17 (59) |  |
| II, A patient with mild systemic disease | 12 (22) | 22 (51) | 5 (12) | 8 (28) |  |
| III, A patient with severe systemic disease | 11 (20) | 4 (9) | 0 (0) | 4 (14) |  |
| **Epidural hematoma (%)** | 5 (12) | 4 (10) | 9 (23) | 10 (40) | 0.01 |
| **Acute subdural hematoma (%)** | 17 (41) | 22 (55) | 15 (39) | 15 (60) | 0.21 |
| **Cerebral contusions (%)** | 18 (43) | 23 (58) | 17 (45) | 13 (52) | 0.54 |
| **Compressed Basal Cisterns (%)** | 21 (50) | 18 (45) | 21 (54) | 7 (29) | 0.26 |
| **Traumatic subarachnoid hemorrhage (%)** | 5 (12) | 3 (8) | 5 (13) | 3 (12) | 0.87 |
| **Midline shift (%)** † | 38 (68) | 40 (87) | 29 (71) | 9 (24) | <0.001 |
| **ICP monitoring during hospital admission (%)** | 43 (77) | 39 (85) | 34 (83) | 22 (56) | 0.01 |
| **IMPACT Unfavorable Outcome (median [IQR])** ‡ | 61 [45, 77] | 69 [46, 78] | 70 [52, 78] | 61 [46, 73] | 0.66 |
| **IMPACT Mortality (median [IQR])** ‡ | 42 [28, 59] | 51 [30, 64] | 57 [33, 62] | 45 [32, 62] | 0.88 |

*Abbreviations*: ASA, American Society of Anesthesiologists; GCS, Glasgow Coma Scale; ICP, intracranial pressure; IMPACT, the International Mission for Prognosis and Analysis of Clinical Trials in Traumatic Brain Injury; IQR, interquartile range; No, number; TBI, traumatic brain injury

^a^Centers with <8 inclusions were excluded from the table. Columns indicate the treatment preference of centers for a larger DC.

† Defined as midline shift more than 5 mm.

‡ Scores give the probability of an unfavorable outcome or death at 6 months postinjury
