# Supplementary material for: The effectiveness of decompressive craniectomy size in traumatic brain injury; an international, observational, comparative effectiveness study
Source: Brain Spine. 2026 Apr 3;6:106019. doi: 10.1016/j.bas.2026.106019 (PMC13090328; doi:10.1016/j.bas.2026.106019)
Supplement: Multimedia component 3 [file mmc3.docx]

**Supplemental Table 3 | Contingency table containing crossovers between absolute DC length categorization and skull-adjusted classification.** The table shows the distribution of number of DC’s which are, for example, categorized as small in absolute value but intermediate or large in skull-adjusted classification.

| **Classification measure** | Skull-adjusted DC ratio | | | |
| --- | --- | --- | --- | --- |
|  |  | *Small* | *Intermediate* | *Large* |
| Absolute DC size | *Small* | 24 | 17 | 2 |
|  | *Intermediate* | 17 | 53 | 16 |
|  | *Large* | 2 | 15 | 26 |

*Abbreviations:* AP, antero-posterior; DC, decompressive craniectomy
